# Exploring the Experiences of Psychiatric Nurses During Care of Patients with COVID-19

**DOI:** 10.1192/j.eurpsy.2022.952

**Published:** 2022-09-01

**Authors:** I.L. Birtalan, O. Kelemen

**Affiliations:** 1 ELTE Eötvös Loránd University, Doctoral School Of Psychology, Institute Of Psychology, Institute Of Health Promotion And Sport Sciences, Budapest, Hungary; 2 University of Szeged, Albert Szent-Györgyi Medical School, Department Of Behavioral Sciences, Szeged, Hungary

**Keywords:** Covid-19, Psychiatric Nursing, Interpretative Phenomenology Analysis

## Abstract

**Introduction:**

The global coronavirus outbreak was viewed as a severe threat to healthcare providers, particularly nurses. COVID-19 has numerous public health management dimensions, including the reorganization of health care workers to support and assist patients.

**Objectives:**

This study used a qualitative approach to gain an insight into the experiences of psychiatric nurses who were treating quarantined patients at various hospitals. This research aimed to investigate the experiences of reassigned psychiatric nurses during the COVID-19 outbreak in Hungary.

**Methods:**

Using a phenomenological approach, we enrolled 7 nurses who provided care for COVID-19 patients from July 2020 to April 2021. The interviews were conducted face-to-face in the form of semi-structured interviews and were analysed using a health-psychology approach: interpretive phenomenology analysis.

**Results:**

Our study shows that pandemic public health reorganization creates novel situations and issues that nurses are forced to address. Our findings suggest that three themes emerge from the data to describe psychiatric nursing: (1) Usage of earlier clinical experiences, (2) Recognizing mental issues, (3) Social networks.

**Conclusions:**

This study suggests professional self-concepts and job satisfaction in relation to treating quarantine patients are affected by the identity and conflicts of psychiatric nursing in a novel situation.

**Disclosure:**

No significant relationships.

